# Implementation of the Canadian National Standards in Breast Cancer Surgical Care: Gaps, Barriers, Enablers and Opportunities

**DOI:** 10.3390/curroncol28010056

**Published:** 2021-01-19

**Authors:** Angel Arnaout, Anubha Prashad, Nadine Dunk, Jess Rogers, Christian Finley, Annemarie Edwards, Mary Argent-Katwala

**Affiliations:** 1 Canadian Partnership Against Cancer, 145 King Street West, Toronto, ON M5H 1J8, Canada; Anubha.Prashad@partnershipagainstcancer.ca (A.P.); Nadine.Dunk@partnershipagainstcancer.ca (N.D.); jess.rogers@partnershipagainstcancer.ca (J.R.); Christian.Finley@partnershipagainstcancer.ca (C.F.); Annemarie.Edwards@partnershipagainstcancer.ca (A.E.); mary@notcontrary.com (M.A.-K.); 2 Department of Surgery, University of Ottawa, Ottawa, ON K1H, 8L6, Canada; 3 Department of Surgery, McMaster University, Hamilton, ON L8S 4L8, Canada

**Keywords:** breast, surgery, standards, cancer, implementation, barriers, enablers

## Abstract

**Background**: Diagnosis and surgical treatment decision making for breast cancers has become increasingly complex. Recently, Canadian Partnership Against Cancer (CPAC) published pan-Canadian evidence-based surgical standards for the care of breast cancer patients. This study was undertaken to assess the degree to which these standards were currently met in practice and to further understand the determinants of their implementation nationally. **Methods**: This study was undertaken in two parts—(1) National survey of breast cancer surgeons to assess the perceived extent of implementation of these standards in their institution and province; (2) Formation of a focus group with a representative sample of breast surgeons across Canada to further understand the barriers and facilitators towards future breast standards implementation. **Results**: 35 surgeons participated in the survey: 66% were from community hospitals. There were four categories of standards that were the most significantly lacking across the country—(a) processes related to quality assurance, (b) turnaround time for pathology results (c) psychosocial and health-related support for the breast cancer patient and (d) breast reconstruction for patients undergoing mastectomy. The focus group included participants from all ten Canadian provinces. For each standard, the 134 determinants fell into three main categories—individual physician (n = 27, 20%); organizational (n = 46, 34%), and system (n = 61, 46%). While specific determinants were present for each category, surgical standards were more likely to be implemented in practice if (1) they aligned with organizational priorities standards; (2) the individual physicians or physician groups were accountable to the organization and/or cancer jurisdiction regarding compliance with the standard; and (3) if infrastructure or resources existed within the organization or system for the reliable collection of relevant, meaningful, practice changing data combined with the capability of benchmarking, peer–peer comparisons and timely feedback to the surgeons. **Conclusion**: The results of this study demonstrated variation, barriers and opportunities for the national implementation of CPAC’s breast cancer surgery standards and identified determinants of high-quality breast surgical care delivery.

## 1. Background

Over the last two decades, breast cancer has become one of the primary health concerns of Canadian women where diagnosis and surgical treatment decision making have become increasingly complex. Modern day breast cancer care demands a multi-disciplinary group of physicians from various specialties to offer personalised, integrated care for the patient. In addition, there is no unified national approach that exists for cancer surgical care in Canada. Surgery is not integrated into most provincial cancer bodies and there are no explicit roles to oversee the systematic evaluation and regulation of cancer surgery in most provinces. As a result, there is clear variation in breast cancer outcomes across the country [1,2,3,4,5]. Recently, the Canadian Partnership Against Cancer (CPAC) published pan-Canadian evidence-based surgical standards for the care of the breast cancer patient [6]. These standards highlight not only the required training qualifications of the treating surgeons, but also the necessary resources to consistently deliver proper care in the treatment facilities. In an effort to establish quality improvement in breast cancer surgical care across Canada, the CPAC investigators undertook a two-part study to facilitate future national level implementation of the breast cancer surgical standards—(1) A national survey of breast cancer surgeons to assess the perceived extent of implementation of these evidence-based standards in their institution and province; and (2) Formation of a focus group with a representative sample of breast surgeons across Canada to further understand the barriers and facilitators towards future implementation of the CPAC breast cancer surgical standards.

## 2. Methods

### 2.1. National Survey to Breast Surgeons

#### 2.1.1. Study Design and Development

We developed an institutional ethics-approved electronic survey that assessed the degree of implementation of each item in the breast cancer surgical standards document [6], as perceived by the surgeon. Questions asked the surgeons to rate the extent of standards implementation for (a) their institution and (b) for the province based on a Likert scale consisting of five categories. A description of the study and a request to provide informed consent for a survey was sent out via email membership mail-out from The Canadian Association of General Surgeons (CAGS, approximately 1800 members). Survey reminders were sent out 4 times over a period of 2 months. Surgeons had to be actively treating patients with breast cancer and had to have agreed to participate with electronic informed consent. Access to the survey was directly from the email invitation once electronic informed consent was obtained. There were no incentives provided to the participants to complete the survey. The surveys were administered through an online software, QuestionPro (QuestionPro Inc., Austin, TX, USA). The usability and technical functionality were cognitively tested by two surgeons. The survey participation email included instructions for respondents (e.g., respondents were unable to change their response once submitted; and each visitor was only able to complete the survey once) (please see Supplementary Materials for the Checklist for Reporting Results of Internet E-Surveys (CHERRIES) and content of surveys).

#### 2.1.2. Survey Data Analysis

For each standard, survey respondents were asked to rate the extent of compliance within their centre as well as their province (using a 5-point Likert-type scale). They were also provided with the opportunity to provide text comments throughout the survey. All survey responses were de-identified prior to analysis. Frequencies of response categories were calculated and displayed as stacked bar charts to show the variation in the extent to which each standard was implemented in the surgeon’s institution and province. The target for implementation was established as 70% (i.e., of respondents answering “to a great extent”); this target was selected by the expert panel that contributed to the development of the breast cancer surgical standards.

Based on the results of the survey, we identified the top four categories of standards which demonstrated the lowest degree of implementation either at the institution and/or provincial level. These categories of standards were then discussed at the focus group (Supplementary Materials).

### 2.2. Focus Group to Assess Enablers and Barriers to Implementation of Breast Cancer Surgical Standards

#### 2.2.1. Study Design

The focus group was conducted during the annual Canadian Association of General Surgeons (CAGS) meeting on 13–15 September 2018 in St Johns, Newfoundland. General surgeons who were already attending the meeting were asked by email explaining the study and asking whether they would be interested in attending a breast cancer surgery focus group. From those that agreed, a purposeful and stratified (by Canadian province or territory) sampling strategy was used to select up to 15 surgeons to participate. Informed consent was obtained from all participants as per ethical guidelines prior to any collection of data. The participants were informed that the Ottawa Health Science Network Research Ethics Board (OHSN-REB) has approved this protocol. The Supplementary Materials includes more details around the methods used for the focus group including the COREQ checklist as well as a discussion guide to assess the enablers and barriers faced by the surgeons as well as opportunities in regard to the implementation of each of the standards.

#### 2.2.2. Data Collection and Analysis

Digital recordings from the focus groups were transcribed verbatim, imported into the qualitative data analysis software NVivo (version 1033) and verified by the study team prior to analysis. This qualitative descriptive study used direct content analysis of data from the focus group. We aimed to identify the determinants that impacted the degree of standards implementation. These determinants were categorised as either enablers, barriers or opportunities, and then further categorised into individual- (surgeon), institutional- (hospital or cancer centre), or system-level (regional provincial) determinants. Individual determinants illustrated physician intentions, knowledge, comfort level and routine practices. Organizational determinants captured determinants that were Influenced from hospital or cancer centre structure, policies and priorities, resource forces, management and organizational culture. Finally, system determinants reflected healthcare system structures and processes. We used a deductive approach to qualitative analysis—analyzing the data based on a predetermined coding structure. Initial Categories for Coding Focus Groups were developed, and sub-categories were noted when reading through the focus group transcripts.

Data analysis occurred in 2 steps—coding and theming. First, coding was performed by 4 team members (A.A., J.R., N.D., A.P.) in an independent manner, for each category of standards discussed. The 4 members then met frequently to compare their coding and discuss discrepancies until a consensus was achieved. Theming, a higher level in abstraction, was then performed by grouping determinants that were similar in concept for each category of standards. Opportunities were noted if potential interventions to support the implementation of surgical standards emerged during the process of coding and theming.

## 3. Results

### 3.1. National Survey

A total of 50 surgeons gave informed consent for the survey but only 35/50 (70% completion rate) completed the entire survey. The 35 surgeons represented the following regions—Eastern Canada (n = 4, 11%), Western Canada (n = 9, 26%), Quebec (n = 3, 9%) and Ontario (n = 19, 54%). A total of 46 per cent of surgeons were in their earlier years of practice (0–10 years), 37% in mid-years (11 to 20 years) and 17% in their later years of practice (20+ years). In addition, 66% of surgeons practiced in community hospitals while 34% practiced in academic hospitals. The following demographics information was collected (Table 1).

The results of the survey demonstrated areas in which there were significant gaps in compliance with the standards. The top four categories of standards that were most under-implemented included (a) processes related to quality assurance, to include data collection; (b) turnaround time for breast pathology results (diagnostic biopsies and surgical pathology); (c) psychosocial and health-related support for the breast cancer patient and (d) breast reconstruction for patients undergoing mastectomy. These formed the basis for the discussion in the focus groups. All surveys were analyzed regardless of their completion ratio.

### 3.2. Focus Group

Focus groups included participants from all ten Canadian provinces (Table 2) and lasted 2.5 h. Discussion of the previously mentioned four categories of standards resulted in the identification of 134 determinants (enablers and barriers). For each standard, the determinants fell into three main categories—individual physician (n = 27, 20%); organizational (n = 46, 34%), and system (n = 61, 46%).

#### 3.2.1. A. Surgical Standards Category 1: Quality Assurance Processes to Include Data Collection

##### Enablers

Surgeon motivation and beliefs were identified as key enablers for data-driven quality improvement. There should be alignment of organizational and provincial incentives with data collection initiatives (e.g., if wait times are an organizational priority, there are incentives for collecting data). Some hospitals have dedicated decision support teams for data extraction and analysis with many hospitals investing in large-scale databases such as the National Surgical Quality Improvement Program (NSQIP).

##### Barriers

Quality of the data collected and consistency of the data collection process were reported as significant barriers. There was a general lack of trust in the data collected (due to inconsistent adherence to technical data specifications), a lack of incentives to enable data collection, analysis and reporting, inconsistent understanding of data sources and restricted access to relevant data. Often, the time, funding, and personnel required for routine data collection and reporting is dependent on an individual surgeon’s volunteer time and their own interests.

##### Opportunities

Participants highlighted the use of Continuing Medical Education (CME) credits for quality processes and data entry into large scale databases as opportunities to facilitate data collection for quality improvement. Synoptic (surgical, clinical and pathological) reporting tools and templates have been implemented in several organizations and offer an opportunity to report on an organization’s performance against provincial benchmarks. Coding for captured data needs to align with coding for pay structure (billing codes) and build on commonly and easily accepted data extraction processes. Many surgeons discussed the creation of communities of practice to facilitate peer-to-peer knowledge transfer and building capacity for quality improvement initiatives.

#### 3.2.2. B. Surgical Standards Category 2: Appropriate Turnaround Time for Breast Pathology (7 days for Diagnostic Biopsies and 14 days for Surgical Pathology)

##### Enablers

Surgeons agreed that there should be joint accountability between pathologists and clinicians (radiologist included) to ensure that the organizational infrastructure was present to meet the targets for appropriate turnaround times. For example, triage of urgent cases and requests for biomarker tests can be done through enlisting the patient for presentation at multidisciplinary rounds or presentations, or directly contacting the pathologists involved or to be involved.

##### Barriers

Several private labs and hospital pathology labs were also reported by the surgeons to not have turnaround time targets or consistent mechanisms to expedite diagnostic or surgical pathology reporting.

##### Opportunities

Participants discussed the opportunity to update protocols in labs regarding breast biomarker testing on diagnostic biopsies and pathology turnaround times in addition to setting up of “batch transportation” of specimens to the labs.

#### 3.2.3. C. Surgical Standards Category 3: Support (Psychological, Education, Rehabilitation, Social, Survivorship) to the Breast Cancer Patient

##### Enablers

Many surgeons identified creative partnerships with allied health services as key enablers; alliance with local cancer foundations and cancer survivorship or wellness programs outside the hospital can help. Several hospitals had volunteer programs where patients get matched with a buddy for social support. Regional patient-driven support groups have been effective in ensuring that patients are aware of the various supports available to them in the region or community.

##### Barriers

Funding for psycho-social services is limited and this was identified as a significant barrier to standard implementation. In addition, different provinces have varying insurance plans which do not consistently cover a similar portfolio of psycho-social support services.

##### Opportunities

Opportunities identified included partnering with private organizations to provide subsidized psycho-social care to breast cancer surgery patients. Identification of volunteer groups can further support the provision of psycho-social care. Personnel such as patient navigators can act as conduits for information and resource sharing to breast cancer patients.

#### 3.2.4. D. Surgical Standards Category 4: Access to Breast Reconstruction Post Mastectomy for Breast Cancer Patients

##### Enablers

During the focus group discussions, many surgeons highlighted breast cancer patients as strong advocates for breast reconstruction post-mastectomy. At the individual physician level, breast surgeons who were aware of the importance of breast reconstruction and had knowledge of the indications, patient eligibility criteria, and surgical techniques used by the plastic surgeons were identified as key enablers to advancing breast reconstruction. From an organizational perspective, having dedicated operating room resources for breast reconstruction enabled its implementation. From a system perspective, a collaborative network of plastic and breast cancer surgeons has been identified as a key enabler to support standard implementation.

##### Barriers

Breast surgeons had the impression from their plastic surgery colleagues that breast reconstruction in a cancer patient was difficult to coordinate, more complex and time consuming. The patients often needed care to be offered in a narrow time window (such as for immediate breast reconstruction at the time of mastectomy) in accordance with cancer wait time targets and was often complicated by the need to undergo additional systemic therapy and radiation, both of which can impact negatively on the surgical outcomes. In addition, coordination of simultaneous surgery between plastic and breast surgeons can be difficult, especially when there is no dedicated OR time or an insufficient number of plastic surgeons available in one organisation. It was clear that rural and remote communities, including community hospitals, have limited access to the full array of options for reconstruction.

##### Opportunities

Greater collaboration with plastic surgeon networks and societies can be an opportunity to ensure optimal breast reconstruction access to patients in the community. Funding levers can be in place to promote the implementation of this standard. Patient voices should be utilized to promote awareness and community access to breast reconstruction.

## 4. Discussion

Although the sample size was small, the results of the national survey surgeons identified four categories of CPAC’ s breast cancer surgical standards that were the most under-implemented in their practice. These included processes related to quality assurance, turnaround time for breast pathology results, psychosocial and health-related support for the breast cancer patient, and breast reconstruction for patients undergoing mastectomy. Although there was clearly variability in the degree to which each individual standard was implemented across provinces, these four categories were perceived to be the most significantly lacking across the country overall.

While specific determinants were present for every category of surgical standards; overall there were general themes that were common across all standards and across multiple provinces. Firstly, surgical standards were more likely to be implemented in practice if they aligned with organizational priorities (such as wait time to diagnosis or treatment, performance of multidisciplinary cancer conferences) or already existing national standards that the organization followed (such as pathology standards for breast biopsies that follow the Canadian Association of Pathology standards). Secondly, standards were more likely to be implemented if the individual physicians or physician groups were accountable to the organization regarding compliance with the standard, especially if the organization itself was also accountable to the cancer jurisdiction in terms of compliance as well. An example of this included wait times to diagnosis and treatment, which is being monitored by the cancer agencies; or compliance with clinical pathways developed by the cancer jurisdictions that breast cancer patients should follow. This was especially important if there were financial incentives related to compliance, either at the organizational or at the individual surgeon level. In Ontario, for example, achievement of breast cancer surgeries within specified wait time targets, compliance with clinical pathways and performance on outcome-related indicators is tied to organizational funding from Cancer Care Ontario. This organizational incentive translates into incentives for surgeons as well, if the organization uses this funding to provide more operating room resources for example. This model has certainly been used in other non-cancer disease sites to improve timely access to surgery across Canada (such as hip, knees, cataracts, dental surgeries). Thirdly, standards related to quality improvement processes were more likely to occur if the infrastructure or resources existed within the organization or system for the reliable collection of relevant, meaningful, practice changing data combined with the capability of benchmarking, peer-peer comparisons and timely feedback to the surgeons. There may be an opportunity to leverage the infrastructure for quality improvement that exists in many organizations and provinces with the capacity that is available (either at system or organizational level) to create incentives for proper data collection and reporting. The provincial surgical synoptic reporting initiative in Alberta, for example, has made it easy for surgeons to enter data and receive feedback regarding their practices in comparison to their peers. Synoptic operative reporting tools have been implemented in several jurisdictions which offer the opportunity to report individual performance against provincial comparisons. The American College of Surgeons’ National Surgical Quality Improvement Project (ACS-NSQIP) is a risk-adjusted database that first started collecting data in 2005 with the intent of comparing hospitals (benchmarking) and for hospital-level quality improvement projects [7]. Since then, its popularity has grown from just a few participating hospitals in the United States to more than 708 participating hospitals worldwide, including many hospitals in Canada. NSQIP captures uniform morbidity variables for all operations and calculates expected risk-adjusted morbidity probabilities. While its extensive use in the US has allowed for regional and national collaboration by sharing of robust data related to outcomes in breast surgery [8], this practice is virtually non-existent in Canada and significant opportunities exist to formalize this process to improve breast cancer surgical outcomes nationally.

The focus group discussions also highlighted that better communication to organizations and provinces regarding the existence of CPAC’s breast cancer surgical standards [6], perhaps with linkages to hospital or cancer centre accreditation processes, was seen to be potentially helpful in standards implementation. Cancer agencies could potentially endorse the CPAC standards and take accountability for compliance of these standards.

With respect to achieving standards related to patient needs and support, whether it be psychosocial, nutritional, rehabilitation related, it was suggested that the system could benefit from creative partnerships with private sector healthcare services. It was noted that patient support resources were often the first to be cut during organizational budgetary restraints, and that patients may be open to private healthcare services if they were approved by the hospitals or cancer agencies. Institutions could negotiate discounted incentives on behalf of the patients in order to facilitate access; furthermore, lists of supportive care providers and resources available could be shared amongst institutions within the same province, for example.

Surgical standards, such as that of access to immediate and delayed breast reconstruction, can benefit from the voices of patient advocacy groups and media in their implementation process; these are often underutilized tools that facilitate the prioritization of cancer surgical standards by the provinces and organizations. Many physicians underestimate the importance and influence that healthcare advocacy has on the profession and feel that they lack the skill and leverage to advocate on behalf of themselves, their practices, their patients, and their profession [9]. However, breast and plastic surgeons are uniquely positioned to advocate based on their clinical acumen, personal experiences with patient care, and their position in the healthcare ecosystem value chain. In the US, the American Society of Plastic Surgeons (ASPS) has long recognized the importance of empowering surgeons alongside patients in advocacy work. To this end, for a few years they have hosted the annual ASPS Advocacy Summit [10], where plastic surgeons not only learn firsthand about the federal, state and regulatory issues impacting the specialty and its patients from nationally recognized political experts and members of the US Congress, but also gain knowledge, experience, and tactics relating to advocacy, which could then be applied toward issues important to them and their institution. Attendance to the ASPS Advocacy Summit has been encouraged and marketed also to medical students and residents, the future leaders in the field of plastic surgery. Such platforms for advocacy training may be a useful method to help empower Canadian breast surgeons to implement CPAC’s breast cancer surgical standards across Canada.

This study has some notable strengths. First, this is the first Canadian national evaluation of the determinants of standards implementation in breast cancer surgery. Although the sample size of the surgeon survey responders was small, the surgeon participants had good representation across sex, age and years in practice. Secondly, the qualitative approach undertaken during the focus groups allowed for a detailed analysis of the determinants necessary to inform strategies to implement high-quality breast cancer surgery nationally.

The current study also has some limitations. For the survey responses, the sample size was small and the survey could have selected surgeons with a stronger interest and an already higher existing level of compliance with breast surgical standards. There could also be a response bias from the surgeons when asked about their perception of the degree of standards implementation, and this may have been different from reality. This is especially true when discussing standards implementation at the provincial level, as some surgeons may not have reliable knowledge at this level. For the focus group, surgeon participants were extracted from the list of surgeons attending the CAGS meeting who were interested in participating. Our opt-in sampling strategy may mean that we only captured attendees of the meeting who had the strongest views (either positive or negative) on the topic and who are most passionate about sharing them, meaning that we may have missed some valuable feedback from those who are more impartial. Finally, while breast cancer surgical care is multidisciplinary, not all members of the multidisciplinary breast team were interviewed, such as pathologists, radiologists and plastic surgeons. Incorporating the views of these groups may provide a potentially fuller picture of the determinants to standards implementation in breast cancer surgical care.

## 5. Conclusions

Overall, the results of this study allowed the investigators of CPAC recognize the variation in the implementation of surgical standards across Canada and identify potential enablers, barriers, and opportunities for intervention for the delivery of high-quality breast surgical care. Future work will need to include the promotion and development of quality improvement strategies and effective resource allocation that is aligned with implementation of the CPAC breast cancer standards in order to improve patient outcomes.

## Figures and Tables

**Table 1:** Surgical demographic information for survey participants.

	Percentage (%) of Practice Dedicated to Breast Surgery	Number of Breast Surgeons at Your Centre	Number of Breast Cancer Surgeries Performed in Your Centre Each Year
**Min**	10	2	20
**Max**	90	9	1100
**Mean**	46	5	382
**n**	35	35	35

**Table 2:** Focus group participant demographics.

Gender	Count	Province	Count
**Female**	8	Ontario	5
**Male**	5	British Columbia	2
		Newfoundland & Labrador	2
		Saskatchewan	1
		Quebec	1
		Alberta	2

## Supplementary Materials

The following are available online at https://www.mdpi.com/1718-7729/28/1/56/s1, Table S1: Checklist for Reporting Results of Internet E-Surveys (CHERRIES)*; Questionnaire S1: Pan-Canadian Standards for Breast Cancer Surgery—Compliance Survey; Table S2: Standards with lowest implementation for each institution and province; Table S3: Consolidated criteria for reporting qualitative studies (COREQ): 32-item checklist. Questionnaire S2: Focus Group Questions.
