# Supplementary material for: Implementation of the Canadian National Standards in Breast Cancer Surgical Care: Gaps, Barriers, Enablers and Opportunities
Source: Curr Oncol. 2021 Jan 19;28(1):565–73. doi: 10.3390/curroncol28010056 (PMC7903263; doi:10.3390/curroncol28010056)
Supplement: Supplementary file 1 [file curroncol-28-00056-s001.pdf]

Copyright © Multimed Inc.

CURRENT ONCOLOGY
A Canadian Cancer Research Journal

Supplemental Materials for

Implementation of the Canadian National Standards in Breast Cancer Surgical Care: Gaps, Barriers, Enablers and Opportunities

Angel Arnaout MD[1,2], Anubha Prashad MBA[1], Nadine Dunk PhD[1], Jess Rogers BA[1], Christian Finley MD[1,3], Annemarie Edwards MBA[1], Mary Argent-Katwala PhD[1]

Listing of Supplemental Materials:

Table S1: Checklist for Reporting Results of Internet E-Surveys (CHERRIES)*

Questionnaire S1: Pan-Canadian Standards for Breast Cancer Surgery - Compliance Survey

Table S2: Standards with lowest implementation for each institution and province

Table S3: Consolidated criteria for reporting qualitative studies (COREQ): 32-item checklist

Questionnaire S2: Focus Group Questions

Copyright © Multimed Inc.

Supplemental Table S1: Checklist for Reporting Results of Internet E-Surveys (CHERRIES)*

Item Category	*Checklist Item*	*Explanation*	*Location*
**Design**	Describe survey design	Surveys were sent out via email membership mail-out from The Canadian Association of General Surgeons (CAGS). Surgeons had to be actively treating patients with breast cancer and had to have agreed to participate with informed consent. A convenience sample was used.	Methods section; Page 3
**IRB Approval and Informed Consent Process**	IRB approval	Institutional ethics approval was obtained	Methods section; Page 3
	Informed consent	It was a voluntary survey. The participants were told the length of time of the survey (10-15 minutes). The investigators were named. The purpose of the study was explained in the invitation email. The email also explained that all responses were confidential and anonymous and that reporting would be on an aggregate level only. Consent was indicated when respondents clicking the 'Go to Survey' button from this page.	Methods section; Page 3
	Data protection	Proprietary survey software was used to ensure data protection. No personal information was linked to survey results in any way. The fully de-identified dataset is kept on password protected computers.	Methods section; Page 4
	Development and testing	The survey was developed from the published pan-Canadian evidence-based surgical standards for the care of the breast cancer patient (1) developed by CPAC. The usability and technical functionality was	Methods section; Page 4

Copyright © Multimed Inc.

	cognitively tested by 2 surgeons.	
Open survey versus closed survey	The survey was a closed survey. Access was provided to only those that received the invitation email.	Methods section; Page 3
Contact mode	Participants were contacted by email only.	Methods section; Page 3
Advertising the survey	The survey was not advertised.	Methods section; Page 3-4
Web/E-mail	The surveys were administered through an online software, QuestionPro. The survey was not posted on a Web site. Access to the survey was directly from the email invitation once the patient performed an electronic informed consent.	Methods section; Page 3
Context	The survey was not posted on a Web site.	Methods section; Page 3
Mandatory/voluntary	It was a voluntary survey.	Methods section; Page 3
Incentives	There were no incentives offered.	
Time/Date	Survey reminders were sent out 4 times over a period of 2 months	Methods section paragraph 1.
Randomization of items or questionnaires	Randomization of items/questionnaires was not performed.	Methods section; Page 4<br>Appendix 3: Pages 16-26
Adaptive questioning	Adaptive questioning was not used as the questions required the participants to rate the extent of standards implementation based on a Likhert scale, with the option of adding comments to each answer.	Methods section: Page 3

Number of Items	There were 1 to 4 questions per page depending on the length of the question.	Methods section; Page 4<br>Appendix 3: Pages 16-26
Number of screens (pages)	The questions were distributed over 10 pages.	Appendix 3: Pages 16-26
Completeness check	All survey items were deemed to be mandatory, and respondents prompted to complete outstanding items before leaving the survey page on which the item was contained.	Appendix 3; Page 16
Review step	Respondents were unable to change their responses once submitted.	Methods section; Page 4
Unique site visitor	Each visitor was only able to complete the survey once.	Methods section; Page 4
View rate (Ratio of unique survey visitors/unique site visitors)	The survey was not posted on a Web site (email invitation only).	Method section; Page 3
Participation rate (Ratio of unique visitors who agreed to participate/unique first survey page visitors)	Not applicable	N/A
Completion rate (Ratio of users who finished the survey/users who agreed to participate)	This was the proportion of surgeons who completed the survey, after giving electronic informed consent.	Results section, 1st paragraph.
Cookies used	Cookies were not used because respondents were recruited from an existing sampling frame.	Methods section; Page 3
IP check	IP address was not used to identify duplicate entries.	Methods section; Page 4

Copyright © Multimed Inc.

Log file analysis	N/A	N/A
Registration	Entry to the survey for each participant was via a unique link from the e-invitation.	Methods section; Page 3
Handling of incomplete questionnaires	All questionnaires were analyzed, whether they were complete or incomplete.	Results section; Page 5
Questionnaires submitted with an atypical timestamp	No respondents were removed from the survey for completing the items too quickly.	Results section; Page 5
Statistical correction	No statistical correction was used.	Results section; Page 5

*Eysenbach, G. (2004). Improving the quality of web surveys: the checklist for reporting results of internet e-surveys (cherries). *Journal of medical Internet research*, 6(3)e34 doi:10.2196/jmir.6.3.e34 http://www.jmir.org/2004/3/e34/

Copyright © Multimed Inc.

Supplemental Questionnaire S1: Pan-Canadian Standards for Breast Cancer Surgery - Compliance Survey

The Pan-Canadian Standards for Breast Cancer Surgery seeks to elevate the delivery of breast surgical care in Canada. The document provides high-level guidance and discussion on the foundational resources and requirements that need to be in place to improve cancer surgical care and outcomes. The standards document has been endorsed by the Canadian Association of General Surgeons (CAGS). The purpose of this survey is to assess compliance of the proposed standards of breast cancer surgery at institutions across Canada, as well as at the provincial-level. We kindly ask that you review the standards and assess if each standard is currently being met at your centre and in your province. Please complete all questions prior to survey submission.

This survey should take approximately 10-15 minutes to complete. The survey will close on August 30, 2018. We would like to thank you for your time and participation in helping us assess pan-Canadian compliance. Upon your feedback, we will collate all responses and use this data to assess gaps across the country and refine our implementation strategy.

Noted below are a few helpful tips to complete the survey:

- Each standard will be presented in turn in the survey.
- For each standard, you will be asked regarding the extent of compliance within your center as well as the province.
- You will have the opportunity to also provide text comments throughout the survey.
- Please feel free to save the completed portions of the survey and come back to it at your convenience. Should you experience any technical issues with this survey, please contact us at quality@partnershipagainstcancer.ca to troubleshoot or recover responses.

Copyright © Multimed Inc.

DEMOGRAPHICS

1. Name

2. Province/Territory of Work (please select only one):
    1. British Columbia
    2. Alberta
    3. Saskatchewan
    4. Manitoba
    5. Ontario
    6. Quebec
    7. New Brunswick
    8. Nova Scotia
    9. Prince Edward Island
    10. Newfoundland and Labrador
    11. Yukon
    12. Northwest Territories
    13. Nunavut

3. Name of Hospital(s)/Cancer Center you are affiliated with?

4. Where do you work?
    1. Community Hospital
    2. Academic Hospital

5. What is your primary role?

Copyright © Multimed Inc.

1. Clinician- Surgeon
2. Clinician- other
3. Administrator
4. Researcher
5. Other __________

6. If you are a clinician, what percent (%) of your practice is dedicated to breast surgery?

7. Number of Years in Practice since latest graduation (from residency or fellowship)?

8. Number of surgeons performing breast cancer surgeries in your centre?

9. Approximate number of breast cancer surgeries performed per year in your center?

Copyright © Multimed Inc.

STANDARDS

1. **SURGEON CRITERIA**

1.1 TRAINING AND MAINTENANCE OF COMPETENCIES

Please comment on the extent to which the following standards are being met in your a) institution b) province:

Standards	Your Institution					Province					Comments
	To a great extent	Some what	Very little	Not at all	Don't know	To a great extent	Some what	Very little	Not at all	Don't know	
1.1.1 Currently, a breast surgeon treating breast cancer adheres to contemporary knowledge in the field in accordance with provincial cancer agency standards and national/international guidelines.											
1.1.2 Currently, breast surgeons are mandated to participate in the maintenance of competency in accordance with provincial and national standards specific to general surgery and surgical oncology.											
1.1.3 Currently, the practicing breast surgeons treating breast cancer have formal, complete and certified training in general surgery and/or surgical oncology under the Royal College of Physicians and Surgeons of Canada (RCPSC). For those not trained in Canada, a similar regimented and accredited training program has been completed.											

Copyright © Multimed Inc.

1.1 DIAGNOSIS

Standards	Your Institution					Province					Comments
	To a great extent	Some what	Very little	Not at all	Don't know	To a great extent	Some what	Very little	Not at all	Don't know	
1.2.1 Currently, patients with abnormal breast imaging or a clinically suspicious breast finding are worked up in a timely fashion, where 90% of breast cancer patients, should have a diagnosis or resolution of that concern within 6 weeks of the date of the abnormal imaging											
1.2.2 Currently, evaluation of breast imaging abnormalities should be performed at facilities that provide nationally accredited digital mammography, breast ultrasound and percutaneous imaged guided core biopsy. If a single facility does not offer or is not nationally accredited to carry out all tests, the patient is referred to an appropriately accredited facility.											
1.2.3 Currently, percutaneous needle biopsy of both benign and malignant disease is the expected standard. Primary diagnosis using open surgical biopsy is the exception.<br><br>Where not available, it is the joint responsibility of the region, institution and surgeon to provide appropriate supports and facilitate timely access to services.											

1.3 SURGERY & MANAGEMENT

Standards	Your Institution					Province					Comments
	To a great extent	Some what	Very little	Not at all	Don't know	To a great extent	Some what	Very little	Not at all	Don't know	
1.3.1 Currently, breast cancer surgeries are only be performed in centres that are adequately resourced to provide or facilitate timely diagnostic, pathologic and surgical access to care either in person or virtually.											

Copyright © Multimed Inc.

1.3.2 Currently, all patients with non-metastatic breast cancer are evaluated by a surgeon early in the care process to determine resectability, prior to the initiation of chemotherapy and radiation therapy.			
1.3.3 Patients in all jurisdictions have access to multidisciplinary treatment decision making.			
1.3.4 Currently, surgeons treating breast cancer regularly participate in multi-disciplinary cancer conferences (MCC) via telemedia, virtually or in person.			
1.3.5 Currently, surgeons treating breast cancer have experience or up-to-date training to perform breast conserving surgery with and without image guidance localization, mastectomy, sentinel lymph node biopsy and axillary node dissection.			
1.3.6 Currently, all patients undergoing mastectomy are routinely notified of their reconstructive options. Eligible patients desiring reconstruction have access to timely reconstructive surgery consultation / evaluation (plastics and breast surgeon) where access to reconstruction does not significantly delay time to surgery.			

2. PRACTICE SETTINGS

2.1 ORGANIZATIONAL CRITERIA

Standards	Your Institution			Province			Comments
	Yes	No	Don’t know	Yes	No	Don’t know	
2.1.1 While the components identified in this document need not be in a single centre or location, established formal networks or relationships exist to ensure timely access to those who are suspected to have or are diagnosed with breast cancer.							

Copyright © Multimed Inc.

2.1.2 Currently, surgeons treating breast cancer present at least 80% of all newly diagnosed cancer patients at multidisciplinary cancer conferences and document it as part of the patient record.											
2.1.2 Currently, when neoadjuvant therapy may be indicated, patients are seen and assessed by medical oncology early (within 2 weeks).											
2.1.2 Currently, the initial treatment (surgery performed or systemic therapy and/or radiation therapy started) for 90% of breast cancer patients, should be initiated within 4 weeks of the date of diagnosis. Appropriate referrals should be made as early as possible. It is the joint responsibility of the surgeon, institution, region, and healthcare team to coordinate care in a timely manner and resources should be applied appropriately to ensure timeframes are met.											
2.1.3 Currently, breast imaging reports adhere to standards set by the Canadian Association of Radiologists and include concordance statements with pathology post biopsy.											
2.1.3 Currently, pathology reports adhere to standards set by the Canadian Association of Pathologists for required elements.											
2.1.4 Currently, 90% of initial core biopsy pathology is reported within 7 days to facilitate treatment decision making. ER/PR/her2 is also reported and available on the final core biopsy result in a timely manner to facilitate treatment decision-making.											
2.1.5 Currently, 90% final surgical pathology is reported within 2 weeks of operation to facilitate adjuvant treatment decision making.											
	To a great extent	Some what	Very little	Not at all	Don't know	To a great extent	To a great extent	Very little	Not at all	Don't know	
2.1.6 Currently, breast cancer surgery is performed in institutions that provide or collaborate with appropriate facilities and resources to support breast surgery including:<br><br>• Day surgery / short stay units											

• Anesthetic services including regional and general<br>• Imaged guided localization as per radiology standards (https://car.ca/patient-care/practice-guidelines/)<br>• Specimen radiography for confirmation of lesion retrieval and margin evaluation (https://www.breastsurgeons.org/new_layout/about/statements/)<br>• Nuclear medicine for sentinel node radioisotope injection. Institutions/ centres/provinces/territory should facilitate access to nuclear material for on-site injection if not available in a nuclear medicine licensed facility<br>• Appropriate fresh breast specimen grossing and processing as per CAP guidelines<br>• Access to pathologists with expertise in breast cancer<br>• Timely access to appropriate immunohistochemistry pathologic evaluation<br>• Access to reconstructive resources<br>• Cancer patient navigators/coordinators and cancer support networks.											
2.1.7 Currently, all patients should benefit from formalized partnerships to cancer centers that result in timely access to clinical trials where appropriate.											
2.1.8 Currently, all patients must have timely access to medical oncology services including consultation, initiation and management of:<br>• Hormonal therapy<br>• Chemotherapy<br>• Biologic therapy											
2.1.9 All patients must have access to timely radiation oncology consultation, radiation therapy and facilities that provide:<br>• Whole/partial breast irradiation with or without boost<br>• Regional nodal irradiation<br>• Palliative radiation for bone or											

Copyright © Multimed Inc.

systemic metastasis<br>• Stereotactic radiation for isolated or limited brain metastasis											
2.1.10 Currently, mental health and psychological services for patients are available throughout the diagnosis and treatment course, into survivorship.											
2.1.11 Currently, early access to palliative care services and supports (preferably close to the patient's home) are available.											
2.1.12 Currently, social/family support services must be provided, including awareness of financial and other supportive resources											
2.1.13 Currently, patient education along continuum of care (pre, during, post treatment) into surveillance and survivorship are provided, including modifiable lifestyle factors (e.g. diet, exercise).											
2.1.14 Currently, patients are made aware of rehabilitation supports:<br>• Post local therapy rehab including physical therapy<br>• Lymphedema management<br>• Prosthetic and post mastectomy bra											

3. QUALITY PROCESSES

3.1 DATA COLLECTION AND CONTINUOUS QUALITY IMPROVEMENT

Standards	Your Institution			Province			Comments
	Yes	No	Don't know	Yes	No	Don't know	
3.1.1 Data collection and continuous quality improvement are an integral aspect of provision of breast cancer care at the individual, local, regional and provincial levels and are currently facilitated by those providing breast cancer care.							

3.1.2 There is implementation of a national, data-driven approach to deliver best practice care. Routine data collection on process and outcomes are be systematically and prospectively captured and benchmarked against national and international standards. This includes systematic classification of adverse events, and periodic review of data to allow for self-evaluation and to promote continuous cyclical improvement (through audit and feedback).							

Standards	Your Institution					Province					Comments
	To a great extent	Some what	Very little	Not at all	Don't know	To a great extent	Some what	Very little	Not at all	Don't know	
3.2.1 Institutions and regions that have regional cancer centres do currently support quality processes such that financial barriers are not a limitation to participation in the quality process initiatives.											
3.2.2 Currently, institutional authorities are collecting relevant quality marker data for audit and feedback intervention in collaboration with surgeons.											
3.2.3 Currently, institutions are supporting adequate collection and measurement of patient experience data.											
3.2.4 There is an expectation that techniques and processes of care will change over time. Currently, when adopting new technologies and techniques, active tracking of adverse events and outcomes are completed.											
3.2.5 Currently, patients at high-risk for negative outcomes, in particular those from vulnerable populations, are identified and navigated through appropriate pathways with monitoring of compliance. Engagement with rural, remote and vulnerable populations to identify cultural and geographic barriers and enablers to optimal care do occur.											

Copyright © Multimed Inc.

4. SURVIVORSHIP

Standards	Your Institution					Province					Comments
	To a great extent	Some what	Very little	Not at all	Don't know	To a great extent	Some what	Very little	Not at all	Don't know	
4.1 At the completion of active treatment, a formalized survivorship plan after the acute treatment phase is clearly articulated in transition to the most responsible primary care practitioner(s) outlining recommend surveillance practice.											

Primary Local, Regional and Provincial Contact Information:

Please provide the names and contact information for representatives in your province with the authority and responsibility to improve surgical care at the local, regional and provincial level.

Local

Regional

Provincial

Copyright © Multimed Inc.

Supplemental Table S2: Standards with lowest implementation for each institution and province

ANALYSIS by Institute and Province **PERCENTAGES**

Standard	Your Organization					Your Province				
	To a great extent	Somewhat	Very little	Not at all	Don't know	To a great extent	Somewhat	Very little	Not at all	Don't know
**Category 1: Quality Processes, Benchmarking and Feedback**										
3.1.2 There is implementation of a national, data-driven approach to deliver best practice care. Routine data collection on process and outcomes are be systematically and prospectively captured and benchmarked against national and international standards. This includes systematic classification of adverse events, and periodic review of data to allow for self-evaluation and to promote continuous cyclical improvement (through audit and feedback).	24%	29%	18%	12%	18%	12%	12%	12%	9%	56%
3.1.3 Institutions and regions that have regional cancer centres do currently support quality processes such that financial barriers are not a limitation to participation in the quality process initiatives.	26%	29%	0%	11%	34%	17%	20%	0%	9%	54%
3.1.4 Institutional authorities are collecting relevant quality marker data for audit and feedback intervention in collaboration with surgeons.	26%	20%	14%	20%	20%	12%	15%	15%	15%	44%
3.1.5 Institutions are supporting adequate collection and measurement of patient experience data.	11%	26%	31%	14%	17%	3%	11%	17%	14%	54%
3.1.6 There is an expectation that techniques and processes of care will change over time. Currently, when adopting new technologies and techniques, active	14%	29%	26%	17%	14%	6%	14%	11%	17%	51%

Copyright © Multimed Inc.

tracking of adverse events and outcomes are completed.										
**Category 2: Turnaround Time for Appropriate Pathology**										
2.1.7<br>90% of initial core biopsy pathology is reported within 7 days to facilitate treatment decision making. ER/PR/her2 is also reported and available on the final core biopsy result in a timely manner to facilitate treatment decision-making.	45%	42%	0%	6%	6%	6%	39%	3%	3%	48%
2.1.8<br>90% final surgical pathology is reported within 2 weeks of operation to facilitate adjuvant treatment decision making	46%	29%	14%	9%	3%	12%	27%	3%	3%	55%
**Category 3: Support (psychological, education, rehabilitation, social, survivorship) to the breast cancer patient**										
2.1.13<br>Mental health and psychological services for patients are available throughout the diagnosis and treatment course, into survivorship.	51%	29%	17%	3%	0%	12%	29%	9%	0%	50%
2.1.15<br>Social/family support services must be provided, including awareness of financial and other supportive resources.	47%	35%	18%	0%	0%	15%	29%	9%	0%	47%
2.1.16<br>Patient education along continuum of care (pre, during, post treatment) into surveillance and survivorship are provided, including modifiable lifestyle factors (e.g. diet, exercise).	37%	49%	9%	0%	6%	11%	29%	9%	0%	51%
2.1.17<br>Patients are made aware of rehabilitation supports:<br>Post local therapy rehab including physical therapy<br>Lymphedema management<br>Prosthetic and post mastectomy bra	66%	23%	11%	0%	0%	14%	34%	6%	0%	46%

Copyright © Multimed Inc.

4.1<br>Patients are made aware of rehabilitation supports:<br>Post local therapy rehab including physical therapy<br>Lymphedema management<br>Prosthetic and post mastectomy bra	54%	29%	6%	3%	9%	21%	26%	6%	3%	44%
**Category 4: Breast Reconstruction Access to Patients Treated in Community**										
1.3.6<br>All patients undergoing mastectomy are routinely notified of their reconstructive options. Eligible patients desiring reconstruction have access to timely reconstructive surgery consultation / evaluation (plastics and breast surgeon) where access to reconstruction does not significantly delay time to surgery.	71%	20%	6%	3%	0%	11%	34%	17%	0%	37%

Copyright © Multimed Inc.

Supplemental Table S3: **Consolidated criteria for reporting qualitative studies (COREQ): 32-item checklist**

No	Item	Guide questions/description
**Domain 1: Research team and reflexivity**		
Personal Characteristics		
1.	Interviewer/facilitator	Which author/s conducted the interview or focus group?<br>Drs. A. Arnaout and C. Finley conducted the breast focus group.
2.	Credentials	What were the researcher's credentials? *E.g. PhD, MD*<br>MD, MSc, FRCSC, FACS<br>Dr. A Arnaout: MD, MSc, FRCSC FACS MBA<br>Dr. C Finley: MD and can you ask him?

Copyright © Multimed Inc.

No	Item	Guide questions/description
3.	Occupation	What was their occupation at the time of the study?<br>**Dr. Angel Arnaout**<br>**Expert Lead**, Knowledge Mobilization, Canadian Partnership Against Cancer<br>**Associate Scientist, Cancer Therapeutics Program**<br>Ottawa Hospital Research Institute<br>**Breast Surgical Oncologist and General Surgeon**<br>Ottawa Hospital<br>**Associate Professor, Department of Surgery**<br>University of Ottawa<br><br>**Dr. Christian Finely**<br>**Expert Lead**, Clinical Measures, Canadian Partnership Against Cancer<br>**Thoracic Surgeon,** St. Joseph's Healthcare Hamilton<br>**Associate Professor,** Surgery, CAWAR, Faculty of Health Sciences, McMaster University
4.	Gender	Was the researcher male or female?<br>Dr. A. Arnaout – Female<br>Dr. C Finley - Male
5.	Experience and training	What experience or training did the researcher have?<br>Dr. Arnaout has research experience in knowledge translation and implementation science, as well as mixed methods and health services research.<br>Dr. Finley has experience in health services research at the policy level.
Relationship with participants		
6.	Relationship established	Was a relationship established prior to study commencement?<br>Drs. Arnaout and Finley were involved in establishing and conducting sessions to develop

Copyright © Multimed Inc.

No	Item	Guide questions/description
		the pan-Canadian standards for breast cancer surgery.
7.	Participant knowledge of the interviewer	What did the participants know about the researcher? e.*g. personal goals, reasons for doing the research*<br>The participants were breast surgeons who were invited to participate in the survey. They were aware that the purpose of the focus group was to discuss the categories of surgical standards that were perceived as being the lowest in implementation and to discuss enablers and barriers to their implementation.
8.	Interviewer characteristics	What characteristics were reported about the interviewer/facilitator? e.g. Bias, assumptions, reasons and interests in the research topic<br>A recruitment email was sent to the participants which highlighted that participation was voluntary. Dr. Angel Arnaout was identified as the Principal Investigator. The participants were informed that the Ottawa Health Science Network Research Ethics Board (OHSN-REB) has approved this protocol. Pg. 4 (in accordance with CHERRIES)
**Domain 2: study design**		The study design was in alignment with Checklist for Reporting Results of Internet E-Surveys
Theoretical framework		
9.	Methodological orientation and Theory	What methodological orientation was stated to underpin the study? *e.g. grounded theory, discourse analysis, ethnography, phenomenology, content analysis*<br>Content analysis was stated to underpin the study. In addition, contextual information was provided to the participants around the Canadian Partnership Against Cancer (CPAC) recently publishing pan-Canadian evidence based surgical standards for thoracic, gynecological, breast and colorectal malignancies. In an effort for quality improvement

Copyright © Multimed Inc.

No	Item	Guide questions/description
		within clinical practice and across Canada, focus groups were proposed with a representative sample of surgeons from each disease site to further understand the barriers and facilitators to implementation of the CPAC surgical standards.
Participant selection		
10.	Sampling	How were participants selected? *e.g. purposive, convenience, consecutive, snowball*<br>Convenience sampling. P. 4 (in accordance with CHERRIES)
11.	Method of approach	How were participants approached? e.*g. face-to-face, telephone, mail, email*<br>Email
12.	Sample size	How many participants were in the study?<br>15
13.	Non-participation	How many people refused to participate or dropped out? Reasons? None<br>None
Setting		
14.	Setting of data collection	Where was the data collected? e.*g. home, clinic, workplace*<br>Annual Canadian Association of General Surgeons (CAGS) meeting on Sept 13-16, 2019
15.	Presence of non-participants	Was anyone else present besides the participants and researchers?<br>Canadian Partnership Against Cancer support staff

Copyright © Multimed Inc.

No	Item	Guide questions/description
16.	Description of sample	What are the important characteristics of the sample? *e.g. demographic data, date* Breast surgeons and surgical oncologists, general surgeons for whom a significant practice was in the area of breast cancer, , stratified by Canadian province or territory
Data collection		
17.	Interview guide	Were questions, prompts, guides provided by the authors? Was it pilot tested? Yes, there were questions used to facilitate discussions for the focus group. The questions were reviewed and validated prior to being used for facilitation.
18.	Repeat interviews	Were repeat interviews carried out? If yes, how many? No
19.	Audio/visual recording	Did the research use audio or visual recording to collect the data? Digital recordings from the focus groups were transcribed verbatim, imported into the qualitative data analysis software NVivo (version 1033) and verified by the study team prior to analysis. (See p.4)
20.	Field notes	Were field notes made during and/or after the interview or focus group? During and after the focus group -See p.4
21.	Duration	What was the duration of the interviews or focus group? The focus group lasted 2.5 hours. (See p.4)
22.	Data saturation	Was data saturation discussed? Yes- See p.14

Copyright © Multimed Inc.

No	Item	Guide questions/description
23.	Transcripts returned	Were transcripts returned to participants for comment and/or correction?<br>No
**Domain 3: analysis and findings**		
Data analysis		
24.	Number of data coders	How many data coders coded the data?<br>4
25.	Description of the coding tree	Did authors provide a description of the coding tree?<br>Yes
26.	Derivation of themes	Were themes identified in advance or derived from the data?<br>Themes were derived from the data.
27.	Software	What software, if applicable, was used to manage the data?<br>NVivo- See p.14
28.	Participant checking	Did participants provide feedback on the findings?<br>No
Reporting		
29.	Quotations presented	Were participant quotations presented to illustrate the themes / findings? Was each quotation identified? e.g. *participant number*

Copyright © Multimed Inc.

No	Item	Guide questions/description
		No
30.	Data and findings consistent	Was there consistency between the data presented and the findings?<br>Yes
31.	Clarity of major themes	Were major themes clearly presented in the findings?<br>Yes. See p.9
32.	Clarity of minor themes	Is there a description of diverse cases or discussion of minor themes?<br>Yes. See p. 9 onwards.

Copyright © Multimed Inc.

Supplemental Questionnaire S2: Focus Group Questions

1. Now that you have seen the survey results of the standards, let's focus on the certain standards that show a wide gap between evidence and practice. For this standard X, we would like for each of you to tell us about your practice related to this standard. For example, is this standard something you follow in your day to day practice? Why or why not?

Facilitators

2. Now we are going to talk about the factors in your institution that you feel help facilitate your daily practice. What organizational, managerial, or resource factors help you practice in the way you wish to practice?

3. Now we are going to discuss a different type of facilitator – personal facilitators. When you look at your colleagues, what personal factors, that is, their personalities, beliefs, education, or experiences, do you think help them to optimize their use of standardized, timely assessment and effective interventions?

Barriers

4. We will now shift gears and talk about the barriers that hinder our practice, making it different from our desired practice. What aspects of your institution, its policies and procedures, the management, or other health care workers, act as barriers to you or your colleagues to implement the standards?

5. Now we are going to discuss a different type of barrier – personal barriers. When you look at your colleagues, what personal characteristics, that is, personalities, beliefs, education or experiences, etc., hinder their practice?

Opportunities, Strategies and interventions

6. Now I would like you to switch your thinking and remember some different strategies that you have found useful. What strategies have you been exposed to help implement the standard and and what strategies did not?

7. You have all mentioned many barriers and facilitators to your practice related to this standard X. Keeping these in mind, please share your opinion on what an ideal intervention is best geared toward increasing a clinician use of the standard. What would effective interventions would look like? What would be the target audiences and how would it be conducted? Are there any additional strategies that you think may be useful?
